# A feedforward inhibitory premotor circuit for auditory–vocal interactions in zebra finches

**DOI:** 10.1073/pnas.2118448119

**Published:** 2022-06-03

**Authors:** Philipp Norton, Jonathan I. Benichov, Margarida Pexirra, Susanne Schreiber, Daniela Vallentin

**Affiliations:** ^a^Bernstein Center for Computational Neuroscience Berlin, 10115 Berlin, Germany;; ^b^Institute for Theoretical Biology, Department of Biology, Humboldt Universität zu Berlin, 10115 Berlin, Germany;; ^c^Neural Circuits for Vocal Communication, Max Planck Institute for Ornithology, 82319 Seewiesen, Germany

**Keywords:** sensorimotor integration, HVC, mathematical modeling, electrophysiology, songbirds

## Abstract

During vocal exchanges, hearing specific auditory signals can provoke vocal responses or suppress vocalizations to avoid interference. These abilities result in the widespread phenomenon of vocal turn taking, yet little is known about the neural circuitry that regulates the input-dependent timing of vocal replies. Previous work in vocally interacting zebra finches has highlighted the importance of premotor inhibition for precisely timed vocal output. By developing physiologically constrained mathematical models, we derived circuit mechanisms based on feedforward inhibition that enable both the temporal modulation of vocal premotor drive as well as auditory suppression of vocalization during listening. Extracellular recordings in HVC during the listening phase confirmed the presence of auditory-evoked response patterns in putative inhibitory interneurons, along with corresponding signatures of auditory-evoked activity suppression. Further, intracellular recordings of identified neurons projecting to HVC from the upstream sensorimotor nucleus, nucleus interfacialis (NIf), shed light on the timing of auditory inputs to this network. The analysis of incrementally time-lagged interactions between auditory and premotor activity in the model resulted in the prediction of a window of auditory suppression, which could be, in turn, verified in behavioral data. A phasic feedforward inhibition model consistently explained the experimental results. This mechanism highlights a parsimonious and generalizable principle for how different driving inputs (vocal and auditory related) can be integrated in a single sensorimotor circuit to regulate two opposing vocal behavioral outcomes: the controlled timing of vocal output or the suppression of overlapping vocalizations.

## Significance

During conversations, we frequently alternate between listening and speaking. This involves withholding responses while the other person is vocalizing and rapidly initiating a reply once they stop. Similar exchanges also occur in other animals, such as songbirds, yet little is known about how brain areas responsible for vocal production are influenced by areas dedicated to listening. Here, we combined neural recordings and mathematical modeling of a sensorimotor circuit to show that input-dependent inhibition can both suppress vocal responses and regulate the onset latencies of vocalizations. Our resulting model provides a simple generalizable circuit mechanism by which inhibition precisely times vocal output and integrates auditory input within a premotor nucleus.

## Behavioral Importance of Vocal Turn Taking

A defining characteristic of spoken conversations is the alternating exchange of vocalizations, often with rapid transitions between speakers and minimal overlap of speech (1). This example of vocal turn taking requires precise control of the onsets of vocalizations, with individual speakers typically responding to their conversational partners within ∼250 ms, although average speeds can vary across linguistic cultures (2).

The ability to coordinate vocalizations in an interspersed manner precedes spoken language developmentally and evolutionarily, extending to other species ranging from nonhuman primates to birds and frogs (3). In all cases, vocal interactions generally require perceiving relevant acoustic signals and initiating exact motor commands to generate an appropriate vocal reply. In the case of vocal turn taking, each interlocutor delays or withholds a response while listening to the other. This social form of sensorimotor coordination reduces acoustic overlap, thereby maintaining unmasked signal transmission and detection. Although this behavior is widespread, little is known about how brain circuits flexibly control whether and when to respond to a partner’s vocalizations. Here, we address this question in a combined experimental–theoretical approach, proposing a role for phasic feedforward inhibition in the orchestration of turn-taking interactions.

## Forebrain Control of Coordinated Vocal Timing in Zebra Finches

The zebra finch has served as a tractable model system for studying the neuroethology of developmental vocal learning (4–7). Due to their distributed nucleated brain architecture (8), songbirds are particularly well suited to study the dedicated neural circuits underlying vocal learning and production (9–14). The vocal–motor pathway has been studied extensively to understand the neural mechanisms underlying production of courtship song, which male zebra finches perform in a unidirectional rather than turn-taking manner. Recently, the convergence of behavioral, anatomical, and electrophysiological evidence has indicated that the zebra finch forebrain “song system” is not solely dedicated to the learned performance of complex courtship song, but that the descending forebrain vocal–motor pathway is also involved in controlling the production of acoustically simpler and largely innate affiliative calls, known as “stack” and “tet” calls (9, 15–19).

Zebra finches engage in pair-specific antiphonal exchanges of these short calls, often coordinating them with one another within the context of a larger group (15, 20, 21). This example of vocal turn taking requires precise regulation of call timing relative to the calls of others. In controlled settings, birds can be driven to adapt their call timing to avoid “jamming” (i.e., overlapping with) the calls of another bird or temporally predictable call playbacks. Blocking the influence of the forebrain vocal–motor pathway by lesioning the song system output nucleus RA (robust nucleus of the arcopallium) or through pharmacological inactivation of the directly upstream premotor nucleus HVC (proper name) does not abolish calling, but drastically impairs the temporal precision of call responses and consequently jamming avoidance (16, 17). In these cases, calls are likely initiated at the level of the dorsomedial nucleus of the intercollicular complex (DM) in the midbrain, which is known to generate call-like vocalizations when stimulated (22, 23). Apart from receiving direct input from the forebrain vocal–motor pathway via RA, DM receives reciprocal inputs from the downstream respiratory brainstem and may receive limited or indirect projections from the hypothalamus (24–26). However, it is not known to receive any direct auditory inputs. Therefore, while activity in the DM may produce calls in a manner that reflects the physiological state, it is unlikely to be sufficient for flexibly controlling call timing relative to specific heard calls in the absence of descending influence from the forebrain.

Electrophysiological recordings within the vocal–motor pathway of awake-behaving birds have revealed bursting activity in sparse-firing HVC premotor neurons as well as downstream RA neurons preceding call production (9, 15, 17, 19). Results from intracellular microdrive recordings have implicated HVC inhibitory interneurons in modulating calling-related premotor projection neuron activity. Furthermore, locally blocking GABAergic inhibition within HVC gives rise to stronger and earlier calling-related bursts in HVC premotor neurons and bilateral disinhibition of HVC results in significantly faster call responses to heard calls (17). Here we utilize these previously observed data and performed electrophysiological recordings in HVC as well as an upstream sensorimotor nucleus in awake birds listening to call playbacks in order to provide the empirical basis for a mathematical model of a vocal timing control circuit.

## Modeling a Vocal Timing Control Circuit

The initiation of a vocal reply entails at least two component processes and can be considered an auditory-evoked motor command. When observing stereotyped premotor activity that is time locked to vocal production, how might we disentangle the contributions of auditory input on production-related activity and subsequent vocal timing? In the absence of paired recordings of premotor neurons and their directly upstream auditory afferents in birds vocally responding to heard calls or the direct stimulation of these afferents evoking premotor activity and vocalization, reply initiation can be addressed by decomposing it into two frames of reference: Vocal related and auditory related. After modeling these processes independently, we can simulate their interactions across a range of time lags and compare the results to experimental data.

While the previous experimental results (16, 19) imply the involvement of HVC in controlling the timing of calls in vocal interactions, the external driving forces as well as the exact functional interplay between identified cell types within this circuitry is unknown. In this study we developed a leaky integrate-and-fire (LIF) neuron-based spiking network model composed of HVC premotor and local inhibitory interneurons as well as upstream vocal- and auditory-related input neurons. We then evaluated the plausibility of connectivity profiles and circuit mechanisms in terms of their consistency with experimental observations.

The proposed mathematical model of HVC’s involvement in call perception and vocal timing allowed us to systematically explore multiple components of this vocal circuit: 1) The interplay between call production–related excitatory premotor drive and local inhibition; and 2) the interactions between sensory input during listening and premotor activity that leads to a vocalization. This model provides a flexible framework, enabling the simulation of experimentally less tractable conditions, including the current state of synaptic efficacy, helping us to dissect the roles of specific circuit components in the control of vocal timing. Specifically, the generation of multiple scenarios in which premotor activity occurs at different time points relative to an arriving auditory stimulus enabled us to derive a plausible mechanism for how inhibition regulates call production onset times that proved consistent with subsequent experimental tests based on the model’s predictions.

## Results

### A Spiking Network Model for Call Production–Related Activity in HVC.

First, we asked whether and how the timing of call production–related premotor activity can be regulated within HVC. We developed a spiking network model consisting of leaky integrate-and-fire neurons connected through biexponential current-based synapses (27) with the initial aim of accurately replicating the call production–related activity of premotor neurons and interneurons within HVC (17) on a microcircuit level. Compared to more biophysically realistic Hodgkin–Huxley-type neuron models, LIF models have fewer parameters and are more computationally efficient in numerical simulations. Integrate-and-fire neurons have previously been successfully applied in modeling of HVC activity during song production (28–30). Here, the intrinsic neuronal properties, as well as synaptic weights and time constants, were fit to data from electrophysiological studies of zebra finch HVC (*SI Appendix*, Tables S1 and S2) (30–32).

**Fig. 1.**
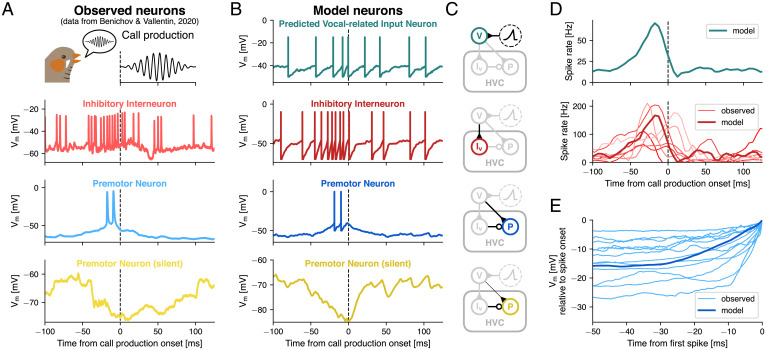
In silico call production–associated neural activity mirrors in vivo data. (*A*) Example membrane potential traces from intracellular recordings of an HVC inhibitory interneuron (red), a bursting HVC premotor neuron (blue), and a silent HVC premotor neuron (yellow) aligned to the onset of a call (dashed line) produced by the observed bird (data from ref. 17). (*B*) Corresponding model traces of an interneuron (red), a bursting premotor neuron (blue), and a silent premotor neuron (yellow), as well as a neuron from a predicted population of upstream vocal-related input neurons (teal, *Top*). (*C*) Circuit diagrams that show model connectivity and highlight the respective populations and their incoming connections. Neuron populations are represented as circles and synaptic connections between populations as lines ending either in excitatory synapses (triangles) or inhibitory synapses (circles). The predicted vocal-related population receives only a transient ramping input current (dashed circle). The silent premotor neuron receives the same input as the bursting premotor neuron; however, excitatory weights from the vocal-related population are lower (8 pA instead of 20 pA). (*D*, *Top*) Spike rate of the predicted vocal-related population, aligned to call production onset (dashed line). (*Bottom*) Spike rate of seven intracellularly recorded interneurons that ramp up in activity prior to call production onset, averaged across trials (light thin lines) and average spike rate of the model interneuron population (dark thick line). (*E*) Ramping subthreshold membrane potential of 12 intracellularly recorded HVC premotor neurons that burst around call production onset (thin light blue lines) and the model premotor neuron (thick dark blue line). All traces were aligned to the time point and membrane potential of their first spike onset (set to zero). Recorded traces were averaged across trials and the model trace was averaged across 100 simulations, each with different randomized amplitude offsets in the input current onto the predicted vocal-related neurons.

Intracellular recordings of identified RA-projecting premotor neurons in HVC [HVC_(RA)_ (17), henceforth referred to as “premotor neurons”) have revealed that they can exhibit a burst of action potentials (2.4 ± 1.2 spikes per burst, mean ± SD; average burst onset: −45 to 33 ms relative to call production onset) or can be hyperpolarized (onset of hyperpolarization: −52 ± 14 ms) shortly before the onset of a produced call (Fig. 1*A*). The model simulated the activity of a representative cell from the set of call production–related bursting premotor neurons and from a set of premotor neurons that do not spike during calling (“silent” with respect to calls) but are hyperpolarized prior to call production onsets (Fig. 1*B*). The activity profile of premotor neurons is modulated by local inhibitory interneurons within HVC (32, 33). During calling, a subset of these interneurons transiently increased their firing rate prior to call production–related premotor bursts, also coinciding with the onset of hyperpolarization in the silent premotor neurons (data from ref. 17) (Fig. 1*A*). The model reproduced this firing rate increase and timing relative to call production (Fig. 1*B*).

In detail, the model consisted of an upstream population of 150 excitatory vocal-related input neurons (34–36) that projected onto both the premotor neuron and a population of 30 local inhibitory interneurons (37) (Fig. 1 *B* and *C*). Similar results were obtained with lower and higher numbers of neurons in those populations, as long as their ratio was on the order of 5:1 (*SI Appendix*, Fig. S1). The predicted vocal-related population was driven by a transient, ramping input current (*SI Appendix*, Fig. S2*A*). The resulting activity led to a transient increase in interneuron spiking (Fig. 1*D*). The main features of the modeled interneuron activity captured the observed range of activity: Simulated population activity peaked at 167 Hz (observed: 64.2 to 210.6 Hz), −17.5 ms relative to call production onset (observed: −32.5 to 7.5 ms) and returned to baseline at 8.1 ms (observed: −15.0 to 52.4 ms). The vocal production–related input to the bursting premotor neuron also replicated the gradual increase in subthreshold membrane potential prior to the burst, which was observed in the intracellular recordings (Fig. 1*E*). The silent premotor neuron was hyperpolarized through inhibitory input from the interneurons. Additionally, it received excitatory input from the vocal-related population, whereby synaptic weights were lower compared to the bursting premotor neuron (*SI Appendix*, Table S2). The longer duration of the hyperpolarization observed in the recorded neurons, compared to the model neuron, might be a result of receiving inhibition from multiple interneurons that reached peak activity at different time points (Fig. 1*D*).

### Feedforward Inhibition as a Mediator of Premotor Activity.

The described network model is biologically plausible but still simple. It consists of only a small number of components and replicates observed call production–related premotor and interneuron activity in the zebra finch HVC. The model is versatile and, considering what is known about the network components, there are several ways in which it could be interconnected. Here, we propose three different model schemes and tested their relative ability to replicate previously observed changes in call production–related HVC activity and experimentally induced perturbations of the circuit.

**Fig. 2.**
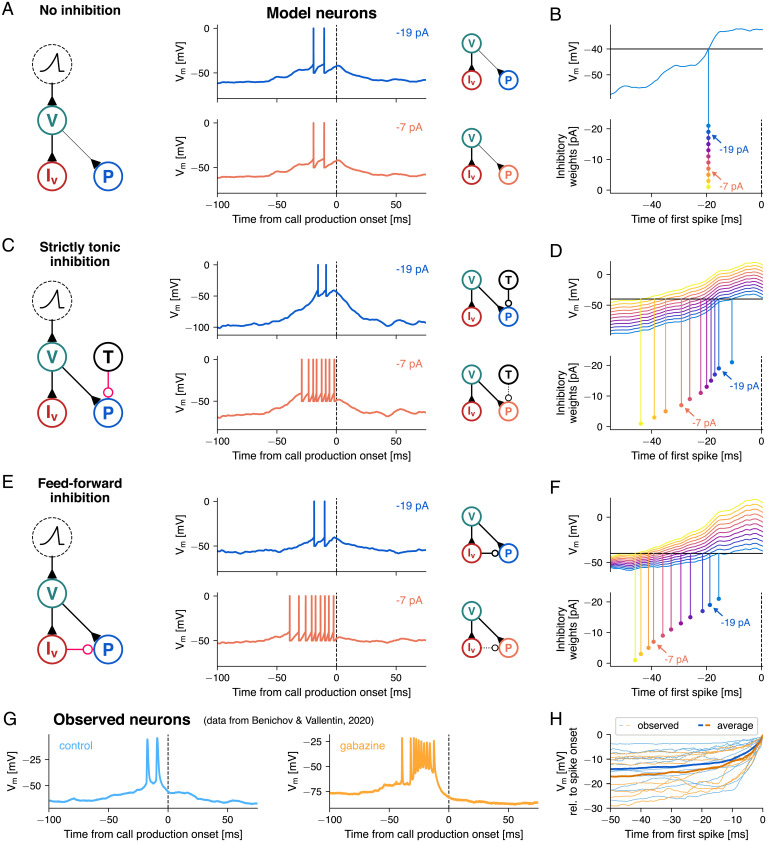
Feedforward inhibition as a mediator of flexible vocal premotor timing. Three alternative models consistent with the intracellular recordings in HVC (Fig. 1), differing in connectivity (*A*, *C*, and *E*). (*A*) In the no inhibition model, local interneurons (“I_v_,” red) driven by vocal-related input neurons (“V,” teal) silence a subpopulation of premotor neurons (shown in Fig. 1 *B* and *C*, *Bottom*). Premotor neurons bursting prior to call production onset (“P,” blue) are triggered by input from the same vocal-related input population as interneurons. The traces show the membrane potential of the bursting premotor neuron when inhibitory weights were −19 pA (blue, *Top*) and −7 pA (orange, *Bottom*). (*B*) Membrane potential traces under a range of inhibitory weights. Arrows highlight the respective traces in *A*. (*C* and *D*) In the strictly tonic inhibition model, the bursting premotor neurons additionally receive inhibition from a population of tonically active interneurons (“T,” black). (*E* and *F*) In the feedforward inhibition model, bursting premotor neurons receive feedforward inhibition instead of solely tonic inhibition, partially balancing the excitatory ramping input. As in *C* and *D*, reduction of inhibitory weights leads to earlier and stronger premotor bursts. (*G*) Two example traces recorded from premotor neurons, one under control conditions (blue, same trace as in Fig. 1) and one after microinfusions of GABA_A_ antagonist gabazine in HVC (orange). Data are from ref. 17. (*H*) Ramping subthreshold membrane potential of the observed HVC premotor neurons that burst around call production onset in the control condition (light blue, *n* = 12) and in after-gabazine microinfusions (light orange, *n* = 8), as well as their respective averages (thick lines).

In the first model, we supposed that inhibition does not play a functional role within HVC during call production (“no inhibition” model, Fig. 2*A*). In the context of learned song production, feedforward excitatory connectivity within HVC can explain the temporally precise sequential activation patterns of premotor neurons, without incorporating local inhibitory influences (11, 12, 38). As some sparsely bursting HVC premotor neurons have been reported to be active during both singing and calling, we decided to first simulate this exclusively excitatory wiring within the context of call production. Because the bursting premotor population in this network configuration was independent of any call production–related inhibitory input from interneurons, it followed that its activity was unaffected by changes in the weights of inhibitory synapses (Fig. 2*B*). Experimentally, however, we found that local disinhibition of premotor neurons through focal application of the GABA_A_ receptor antagonist gabazine resulted in stronger and earlier bursts relative to call production onset (Fig. 2*G*) (17). This discrepancy, together with evidence of the high connection probability between interneurons and premotor neurons in HVC (31, 32, 39), suggested that the no inhibition model was insufficient to explain the call production–related neural activity in HVC.

Next, we tested two models that include inhibition, with a unidirectional local connectivity between interneuron and premotor neuron. As our focus was on the activity that resulted in a premotor burst, as well as the timing of these bursts, possible effects of premotor bursts through recurrent connectivity with interneurons were excluded. A direct inhibitory input to the bursting premotor neurons was added either in a strictly tonic model or a phasic model containing a tonic component (the phasic element was triggered by external inputs, temporarily increasing activity above tonic background activity levels). Both temporal patterns of inhibition are biologically plausible and have been reported to maintain the excitatory/inhibitory balance of a network (32, 40, 41). In HVC, multiple types of interneurons have been characterized (42, 43), exhibiting tonic firing patterns in vitro (44) and structured phasic activity during song production (32). Although production-related increased interneuron activity has been seen preceding calls, the extent to which the effects of disinhibition could also be explained by temporally independent tonic inhibition alone is not clear.

The strictly tonic inhibition model included a population of consistently active interneurons synapsing onto the bursting premotor neuron with adjustable inhibitory weights (Fig. 2 *C* and *D*). In the feedforward phasic inhibition model, interneurons driven by the predicted vocal-related input neurons transiently affected bursting premotor activity depending on the strength of the inhibitory connection (Fig. 2 *E* and *F*).

Both models simulated the activity patterns of premotor neurons and interneurons during call production. We asked how varying inhibitory weights influenced premotor burst onsets, strength, and subthreshold membrane potentials for each wiring scheme. To do so, we simulated the aforementioned local gabazine infusion conditions through progressive reduction of the inhibitory weights on the premotor neuron synapses in 2 pA steps. In both models, premotor bursts occurred earlier and contained more action potentials with reduced inhibition (strictly tonic at −19 pA vs. −13 pA: 2 vs. 4 spikes per burst; feed forward at −19 pA vs. −13 pA: 2 vs. 5 spikes per burst), similar to the results obtained experimentally (control vs. gabazine mean: 2.14 ± 1.10 vs. 5.17 ± 2.40 spikes per burst) (Fig. 2 *C–F*, compare Fig. 2*G*).

The main difference between the strictly tonic inhibition and feedforward phasic inhibition model was apparent in the effects of inhibition on the resting membrane potential of premotor neurons preceding call production–related spiking. In the strictly tonic inhibition model, inhibition acted equally across the entire peri-call interval. Therefore, reducing the weights effectively shifted the baseline membrane potential uniformly toward spike threshold. As a result, the ramping potential reached spiking threshold at successively earlier time points as the inhibitory synaptic weights were decreased (Fig. 2*D*). The strictly tonic inhibition model thus predicted a considerable increase in baseline membrane potential prior to premotor bursts caused by the reduction of inhibition, which was not observed during the experimental perturbation with gabazine (Fig. 2*H* and *SI Appendix*, Fig. S3). In the feedforward phasic inhibition model, the transient increase in interneuron firing counterbalanced the excitatory vocal-related drive during the preburst ramping more sparsely in time. In this case, when reducing inhibitory synaptic weights, we observed a more modest shift in the baseline potential as well as an increase in the steepness of the ramping subthreshold potential, resulting in an earlier threshold crossing and thus earlier and stronger premotor bursts (Fig. 2*F*). These results are comparable to the changes in the membrane potentials observed in the control vs. the gabazine conditions in experimental data from ref. 17 (Fig. 2*H* and *SI Appendix*, Fig. S3).

Taken together, these simulations demonstrate that feedforward connectivity between interneurons and premotor neurons was sufficient to capture the call production–related activity observed in experiments. To assess the model’s robustness against potential perturbations, we simulated a range of synaptic weights and population sizes for the excitatory and inhibitory inputs onto the premotor neurons. We tested the resulting premotor traces for consistency with two features observed in the electrophysiological recordings: A baseline membrane potential between 5 and 25 mV below spike threshold (Fig. 1*E*) and the emission of one to six action potentials in the 50 ms preceding call production (17). Those criteria were fulfilled in a relatively broad range of synaptic weight combinations (*SI Appendix*, Fig. S4) and population sizes (*SI Appendix*, Fig. S1). Reduction of excitatory weights in this feedforward phasic inhibition model could cancel and ultimately reverse the effect of the ramping input, leading to a hyperpolarization of the premotor neuron (Fig. 1*B* and *SI Appendix*, Fig. S4). Reducing the inhibitory weights resulted in both stronger and earlier premotor bursts, supporting the role of HVC interneurons in call timing control.

The principles of our feedforward phasic inhibition model can be found across many brain areas (45, 46). Here, we demonstrated that this type of network can explain the occurrence of vocal output at variable time delays after a fixed vocal–related input, which is regulated by local inhibition. So far, we considered this fixed vocal–related input as the reference point after which a vocalization might occur. However, auditory signals might also influence the production of vocal output. To test whether the same mathematical model can incorporate both auditory- and vocal-related input (i.e., elucidate HVC’s function as a sensorimotor integrator) we next considered an auditory-related input resulting from heard calls as an external driving force onto this circuitry.

### Auditory Input from Heard Calls Evokes Changes in Activity of HVC Interneurons and HVC-Projecting Nucleus Interfacialis Neurons.

To achieve vocal turn taking with minimal overlap, birds must produce a vocalization at appropriate times in response to their vocal partner. The success of this behavior not only relies on the capacity to vocalize but also on the ability to integrate information related to the partners’ calls. We therefore asked whether heard calls evoke activity changes in HVC, independent of vocal production.

**Fig. 3.**
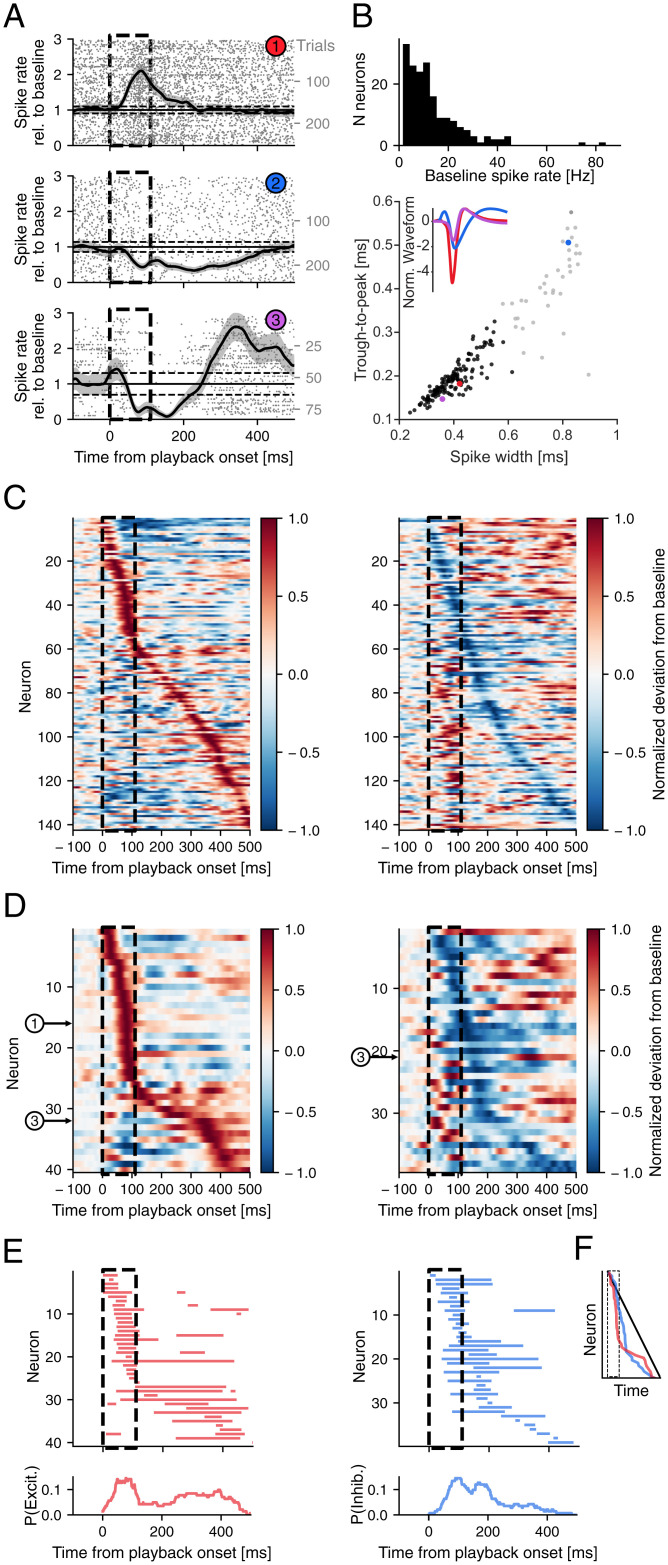
Responses in HVC to call playback stimuli. (*A*) Average spike rate of three HVC neurons in response to call playbacks, normalized to baseline activity. Example of excitatory (*Top*), inhibitory (*Middle*), and mixed response (*Bottom*). Gray patches mark average ± SEM. Horizontal lines mark baseline activity ±2 SD. (*B*, *Top*) Histogram of the average firing rate during the 500 ms prior to playback onset (baseline) of the 179 neurons recorded for a minimum of 20 trials. (*Bottom*) Distribution of mean spike waveform characteristics for each neuron. Black dots are putative interneurons, determined by *k*-means clustering. (*Inset*) Averaged spike waveforms of three neurons corresponding to those displayed in *A*. Waveforms were normalized based on the peak amplitude. (*C*) Average spike rates from the 142 putative interneurons, normalized to baseline (0) and absolute maximum deviation from baseline (1 or −1), aligned to call playback onset and sorted by time of maximum positive (*Left*) or negative deviation (*Right*) after playback onset. Dashed black lines mark time of call playback. (*D*) Subsets of neurons that show significant excited (*Left*) and/or inhibited responses (*Right*) after playback onset, sorted by maximum of positive or negative deviation, respectively. The numbered circles highlight the example neurons shown in *A*. (*E*) Significant intervals of increased (*Left*) and decreased (*Right*) activity per neuron, sorted as in *D*. (*Below*) Probability distribution of responsiveness of putative interneurons that are excited or inhibited, respectively, at each time point. (*F*) Time of the sorted positive (red) and negative (blue) extrema as seen in *D*, compared to the values expected if extrema were distributed uniformly in time (black diagonal line), i.e., independent of playback.

To this end, we extracellularly recorded neurons in HVC of awake, head-fixed (and as a consequence, vocally unresponsive) adult male zebra finches (*n* = 225 neurons, *n* = 4 birds) while presenting a set of call playbacks. This awake and head-fixed setup allowed us to record auditory-evoked responses in HVC that are not confounded by activity directly related to vocal production. To characterize the call playback–related activity profiles of the recorded neurons, we only took units into account that were recorded during the presentation of at least 20 playbacks (179/225 units; Fig. 3 *A* and *B*).

We hypothesize that our neural recordings during call playbacks were oversampling the activity of HVC interneurons for several reasons. First, sparse bursting activity of premotor neurons in adult zebra finches only occurs during song or call production (9, 17). Second, HVC projection neurons have been shown to be unresponsive to song playback in awake adult zebra finches (47), whereas interneurons increase their activity in response to the tutor song presentation (13). Third, the baseline firing rate of the 179 recorded neurons was on average 13.66 Hz ± 12.05 Hz (minimum: 1.53 Hz, maximum: 84.02 Hz; Fig. 3 *B*, *Top*), which is similar to what has been reported for HVC interneurons previously (48) and generally higher than HVC projection neuron firing (premotor: ∼0 Hz, other projection neurons: 1.5 ± 0.5 Hz).

To further restrict our analysis to putative interneurons, we relied on classically defined spike waveform features (49). We classified neurons as putative interneurons or projection neurons (Fig. 3 *B*, *Bottom*). This analysis showed an overrepresentation of neurons (142/179 units) with fast (trough to peak = 0.1955 ± 0.0422 ms), narrow waveforms (full width at half maximum = 0.4005 ± 0.0749 ms), and high baseline firing rates (15.21 ± 12.51 Hz), characteristic of interneuron populations (Fig. 3*B*, marked in black after *k*-means clustering) (50).

Next, we aligned the activity of the putative interneurons to call playback onset. Sorting the neurons based on either the maximal or the minimal firing rate suggested that activity changes can disproportionally occur during the call playbacks (Fig. 3*C*). To further analyze this temporal distribution, we identified the putative interneurons that showed significant changes in activity (*SI Appendix*, *Materials and Methods*) after the onsets of call playbacks. We found 59/142 putative interneurons to be significantly responsive to call playbacks (Fig. 3 *A* and *D–F*) and were able to distinguish three general response patterns among these neurons: Excitatory responses with one or more intervals of significantly increased activity (*n* = 20/59 neurons), inhibitory responses with one or more intervals of significantly decreased activity (*n* = 19/59 neurons), and mixed responses with intervals of both significantly increased and decreased activity (*n* = 20/59 neurons) (Fig. 3 *A* and *E*) in response to call playback.

When sorting the interneurons that showed an excitatory or mixed response by the time of their maximal firing rate we observed an overrepresentation of increased firing during the playback interval (Fig. 3 *D*, *E*, *Left*, and *F*, red). If increases in activity were uniformly distributed within the 500 ms following playback onset (Fig. 3*F*, black), on average 22% of cells would be expected to increase activity during the 110-ms playback interval. We found, however, that 31/40 (77.5%) of these cells significantly increased their activity during the playback interval. Similarly, for interneurons that showed inhibitory or mixed responses, 24/39 (61.5%) began to decrease their activity during the playback interval (Fig. 3 *D* and *E*, *Right*). Of the 20 cells that exhibited mixed responses, 12/20 were first excited and then inhibited, whereas 8/20 were first inhibited and then excited. Together, these data suggest that calls of a vocal partner can provide fast excitatory inputs onto HVC, which can drive increases as well as decreases in HVC interneuron activity.

**Fig. 4.**
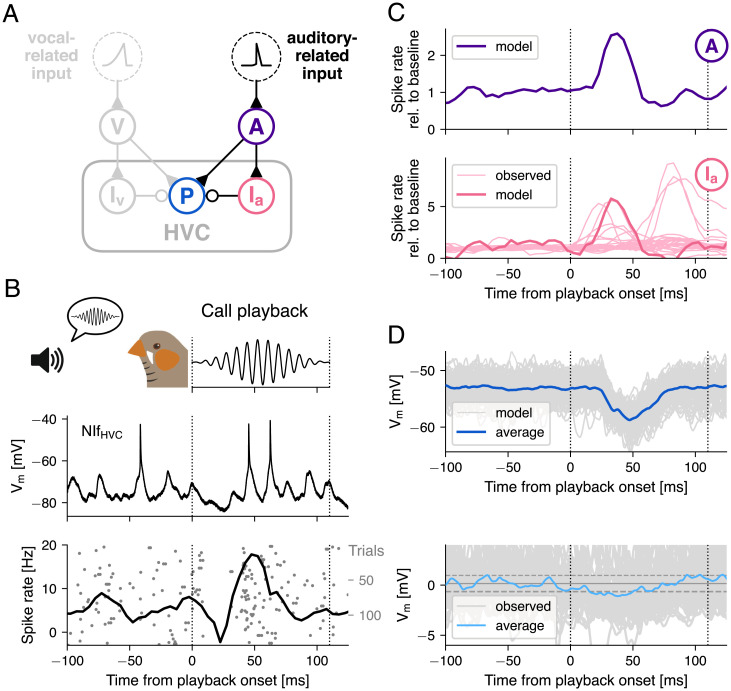
Auditory-related input onto HVC from sensorimotor nucleus NIf. (*A*) Circuit diagram of the feedforward inhibition model, expanded with an auditory-related input population (“A,” purple) and a second inhibitory population (“I_a_,” pink) providing excitatory and feedforward inhibitory input to the premotor neuron (“P,” blue), respectively. (*B*) Intracellularly recorded membrane potential trace of an HVC-projecting NIf neuron that showed a peak in activity after call playback (*Top*). Spike raster and peristimulus time histogram of the same neuron across 143 trials (i.e., playbacks; *Bottom*). (*C*) Population activity of the model auditory-related input population (*Top*), which receives a short, ramping input current (*SI Appendix*, Fig. S2) after call playback onset (dotted line). This triggers a peak of activity in the interneuron population (*Bottom*) that is consistent with putative interneurons recorded extracellularly in HVC (neurons that significantly increased their activity within 100 ms after playback onset; light pink). (*D*) The bursting premotor neuron (“P,” blue in *B*) at rest (i.e., while not receiving vocal-related input from V and I_v_) is transiently hyperpolarized. Model traces from 100 simulations with different randomized input currents (gray) and the average (blue). (*Below*) Intracellular recordings of an example premotor neuron aligned to playback onset (dotted line), which is significantly hyperpolarized following playback onset. Horizontal lines show mean baseline potential ± 2 SD (baseline: −100 to 0 ms).

To further understand the auditory signals that contribute to call playback–related activity in HVC (Fig. 4*A*), we investigated its main source of higher auditory input: The nucleus interfacialis (NIf) (34, 51). To this end, we performed intracellular recordings of NIf neurons (*n* = 6 identified HVC projection neurons and 1 nonidentified NIf neuron) while presenting call playbacks to adult, awake, headfixed birds (*n* = 6 birds). These NIf_(HVC)_ neurons displayed call playback–related activity represented by either a suppression (−2.6 ± 3 Hz delta from baseline (silent period 300 ms prior to playback) or increase in firing rate (5.6 ± 6.6 Hz delta from baseline) in response to call playbacks (Fig. 4*B*). Call playback–evoked activity in NIf (mean: 60.86 ± 24.31 ms; see also ref. 52) had a similar or earlier latency to HVC responses (mean: 114.57 ± 115.59 ms). Because HVC neurons receive direct inputs from NIf (37), and auditory responses as well as spontaneous activity in HVC are significantly reduced after NIf lesions (53–55), we hypothesized that the observed NIf activity contributes to call playback–related changes observed in HVC.

In order to account for the observed auditory-evoked activity changes in HVC, we mirrored the feedforward phasic inhibition model (Figs. 1 and 2*E*) to include an upstream excitatory “auditory” population and a local interneuron population. These were connected through the same circuit motif of excitation and feedforward inhibition (Fig. 4*A*). Based on the synaptic delay of auditory input previously reported in HVC (56), which closely corresponded to the observed activity profile within NIf (Fig. 4*B*), the auditory population received a shorter ramping input current that peaks at 35 ms after playback onset with a short quadratic upstroke and linear downstroke (*SI Appendix*, Fig. S2*B*). In the model interneuron population, this led to a transient peak in activity that matched the observed activity of an “early” peaking subset of the recorded putative interneurons (Fig. 4*C*). The experimental data also included interneurons with later peaks in activity, which the model could not account for with the given input current. However, diverse patterns of auditory responses to calls in NIf, including “late” and “continuous” increases in activity, have been reported (52). These responses might provide the necessary input to explain later changes in HVC activity. In contrast to the original model (Fig. 2*E*), the balance of synaptic weights from the auditory-related input is biased toward inhibition, so that the premotor neuron in the absence of input from the original vocal-related population was transiently hyperpolarized after call playback. Examples of hyperpolarization were indeed observed in experimental data upon realigning intracellular recordings of premotor neurons to call playback onsets (17) (Fig. 4*D*). However, these hyperpolarizations were lower in amplitude than those produced by the model. This may be the result of the model’s current-based synapses, which do not account for the chloride reversal potential. This reversal potential is close to the resting membrane potential of premotor neurons (32). Therefore, the amplitude of inhibitory postsynaptic potentials is likely overestimated in the model when the membrane potential approaches this reversal potential. Additionally, we observed cases of premotor neurons that were slightly depolarized in the same time frame (*SI Appendix*, Fig. S5). This depolarization would be expected in cases for which inhibitory input is sufficiently low relative to excitation from the auditory input population, a scenario we explore later in more detail.

### Integrating Auditory- and Vocal-Related Control Mechanisms.

**Fig. 5.**
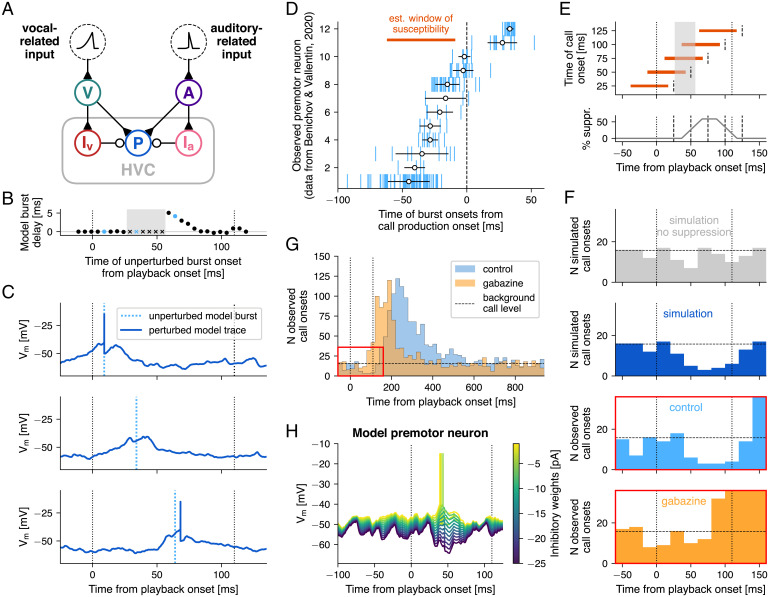
Playback-evoked inhibitory suppression of premotor activity can reduce call overlap. (*A*) Circuit diagram of the full model. (*B*) Simulation of the interaction between precall premotor activity (ramp and burst) and playback-evoked inhibitory suppression at different relative time points. Premotor bursts can be suppressed (marked by crosses) or delayed (*y* axis) by playback-induced inhibition when the premotor burst occurs at different time points (*x* axis) relative to the playback onset (left dotted line). The gray rectangle marks a time window during which premotor bursts are suppressed. The blue dots and cross mark the burst onset times of the example traces shown in *C*. (*C*) Three example traces from the premotor neuron, receiving vocal-related input at different times relative to call playback. (*Top*) Burst occurs before peak in inhibition and is therefore not perturbed relative to the burst onset without inhibitory suppression (blue dotted line). (*Middle*) Burst is suppressed as preburst ramp occurs during inhibitory suppression, preventing the membrane potential from reaching spike threshold. (*Bottom*) Ramp is modified by inhibition, but potential still reaches threshold after a delay. (*D*) Timing of all burst onsets during multiple trials relative to onset of call production (*x* axis) for all 12 observed HVC premotor neurons in the control condition (*y* axis). The orange bar marks the estimated time window during which premotor neurons triggering call production are susceptible to inhibitory suppression. (*E*) Five example call onsets (dashed lines) with their associated windows of susceptibility (orange bars). Below is a function of the percentage of overlap of the window of susceptibility with the suppression window (gray rectangle, as in *B*), i.e., the percentage of premotor suppression (*y* axis) against the onset time of a hypothetically produced call. Example call production onsets from above are marked by dashed lines. (*F*) We simulated a random uniform distribution of call production onsets (gray). We then removed calls with a likelihood proportional to the suppression function in *E*. The resulting call production onset distribution (blue) matches that of the behavioral experiments in the control condition (light blue), both of which show a dip in call likelihood between around 50 and 110 ms after playback onset. Consistent with our model, bilateral gabazine infusions in HVC abolished that dip (orange). Instead, response likelihood begins to sharply increase around 80 ms after playback onset. Histogram bars after 100 ms are cut off in the gabazine panel. (*G*) Full histograms of call production onsets from five birds during control (blue) and gabazine conditions (orange) across the 1-s interplayback interval. The red rectangle highlights the section depicted in *E*. The horizontal dashed line marks the background calling level in the control condition (15.75 calls per 20-ms bin on average between 600 and 1,000 ms after playback onset). (*H*) Reduction of inhibitory weights onto the model premotor neuron reverses its playback-induced hyperpolarization (Fig. 4*D*), ultimately eliciting a spike. Data in *D*, *F*, and *G* are from ref. 17.

Next, we wanted to investigate the interaction between call production–related premotor drive and auditory-evoked inhibition by incorporating both within the model (Fig. 5*A*). We were then able to simulate call production at different time points relative to a heard call. To examine whether auditory input might lead to vocal suppression, we varied the time difference between the input currents to the vocal-related and the auditory populations while observing the delay or the suppression of bursts caused by auditory-evoked inhibition. Between 25 and 55 ms after playback onset, bursts were suppressed, as playback-evoked inhibition prevented the neuron from reaching spike threshold (Fig. 5 *B* and *C*). Bursts which occurred earlier were unaffected, while later bursts were delayed by up to 5 ms, due to a perturbation of the preburst ramp in subthreshold potential.

After we determined the time window following playback onset, during which premotor bursts were suppressed (“suppression window”: 25 to 55 ms), we estimated a time window prior to the onset of call production, during which the suppression of premotor bursts can potentially cancel an imminent call (“estimated window of susceptibility”). Average burst onset times of observed premotor cells varied between −45.0 and +33.4 ms, relative to onset of call production (data from ref. 17) (Fig. 5*D*). We set the start of the estimated window of susceptibility to 60.95 ms before call production onset (mean − SD of the earliest average burst onset) (Fig. 5*D*). The end of the window was defined as 10 ms prior to call production onset, with the assumption that after this time point any further changes at the level of HVC could no longer influence call timing, as downstream motor commands would have already been initiated.

Relating the suppression window to the estimated window of susceptibility allowed us to predict the behavioral outcome of the proposed suppression mechanism, given two assumptions regarding the temporal distribution and function of the precall premotor drive. First, we assumed that bursts were distributed nearly uniformly across time before call production onset. Such a distribution has long been hypothesized for premotor neurons during song production (e.g., refs. 9, 11, 57) and more recently received support from electrophysiological recording and imaging of large populations of HVC projection neurons (58, 59). Despite a smaller dataset, intracellular recordings during call production suggested a similar distribution for precall activity (Fig. 5*D*). Second, we assumed that the triggering of a timed call response depends on the number of premotor spikes. Call-like vocalizations can be elicited by electrical stimulation of downstream nucleus DM (22, 60, 61). It is likely that excitatory input to DM from HVC (via RA) is sufficiently strong to elicit a call response. Suppression of a significant number of premotor bursts through auditory-evoked inhibition would thus reduce the likelihood of a call response.

Given these data-driven assumptions, the amount of overlap between the suppression window and the estimated window of susceptibility predicted the likelihood of a call being triggered. We determined the overlap as a function of call production onset timing relative to playback onset (“suppression function,” Fig. 5*E*). Based on previously observed population-level call response distributions (16, 17), we assumed that the peak in response onset times arises out of a low but nonzero baseline level of calls that are not produced as replies, per se, to a given playback and are therefore uniformly distributed in time, relative to playbacks. We hypothesized that playback-evoked inhibition would resulted in a dip in the call production onset distribution shortly after the onset of playbacks but before the rising peak in call reply onsets. We first generated uniformly distributed random call production onset times (Fig. 5*F*, gray), reflecting the low level of incidental background calling (horizontal dashed lines). To simulate playback-evoked call suppression, we then removed individual calls with a probability proportional to the suppression function (Fig. 5*F*, dark blue and *Materials and Methods*). For our model we decided to assume a linear relationship between the degree of suppression and the probability of call initiation. Similar results were obtained when considering a nonlinear relationship (*SI Appendix*, Fig. S6).

Through this process we effectively simulated the behavioral output (i.e., call onset response time distribution) predicted by the modeling of fast and transient auditory-evoked inhibitory suppression of premotor activity. According to the prediction, call likelihood decreased between 50 and 110 ms postplayback. Inhibitory suppression in the model had the potential to suppress call production shortly after an incoming auditory cue and could thereby partially reduce the overlap of calls between two vocally interacting birds. Complete overlap of calls (i.e., two birds initiating a call within 50 ms of each other) was not affected, as in this case the initiation of each call would occur before auditory information about the partner’s call affects activity in HVC.

For a comparison to observed behavioral data (17), we pooled the call production onset times of 5 adult male birds responding to regularly timed call playbacks (one call per second) in either a control condition or after gabazine application (Fig. 5*G*). The onset of call suppression in the predicted call production onset distribution matched that of the control condition (Fig. 5*F*). At around 150 ms after playback onset, the observed call responses sharply increased above the presuppression baseline. At this point the predicted distribution deviated from the recorded distribution, as increased call likelihood in response to the playback was not factored into this simulation. In the gabazine condition, no reduction of call responses following playback could be observed (Fig. 5 *F* and *G*). This outcome was expected, as a reduction of inhibitory efficacy in HVC reduced or even eliminated the effect of the proposed suppression mechanism. Instead, response likelihood increased above baseline between 80 and 90 ms after playback onset, i.e., already before playback offset.

### Inverting Excitatory/Inhibitory Balance Leads to Auditory Triggering of Premotor Activity Instead of Suppression.

In order to test whether the full model (Fig. 5*A*) can account for the increased likelihood of fast responses, we replicated the gabazine-induced disinhibited state by reducing feedforward inhibitory weights. While reducing vocal production-related inhibition could partially account for the reductions in call response latency observed in the gabazine experiments, premotor bursts occur, at most, 50 ms earlier (Fig. 2*F*). It did not fully explain the observation of the largest time differences in the case of the fastest responses (the most extreme bird reduced its response latency by 200 ms after gabazine application (17)). We wondered how auditory input might contribute to the initiation of the fastest call responses during gabazine treatment. Therefore, we gradually decreased the synaptic weights of the auditory-driven interneuron population onto the premotor neuron, mimicking the effects of gabazine application. As the inhibitory weights decreased, the excitatory drive of the auditory population increasingly dominated the synaptic input, leading to a transient depolarization in the premotor neuron (Fig. 5*H*). With inhibitory weights below 6 pA the auditory input elicited premotor spiking. This inversion from suppression to firing in premotor neurons could potentially recruit a larger population, projecting stronger premotor drive along the vocal motor pathway, resulting in earlier vocal responses. This larger pool of projecting premotor neurons may also explain the increased variability in acoustic features of calls produced during bilateral gabazine infusion (17). Together with the lifting of auditory suppression, the model can account for the increase in short latency vocal responses via the removal of feedforward inhibition driven by vocal- and auditory-related premotor inputs.

Taken together, these results indicate that inhibition within HVC regulated the behavioral output on two time scales: On a short time scale, an auditory-evoked increase in inhibition led to a suppression of vocal motor output while the social partner was producing a vocalization and, thus, a call was withheld and vocal overlap was reduced. On a longer time scale, inhibition was related to premotor preparation and controlled the precise timing of a vocalization.

## Discussion

We developed a phasic feed–forward inhibition network model of zebra finch HVC that illustrates how cortical control over innate vocalizations (calls) can facilitate vocal turn taking. In the proposed model, HVC integrates auditory and premotor information to gate call production.

The model accounts for the observation that the restriction of inhibitory influence in HVC leads to birds responding significantly faster to the calls of a vocal partner (17). This reduction in response latency can be brought about by the shift in the balance of excitatory and inhibitory input onto model premotor neurons. First, the dominance of excitation during the integration of vocal-related input causes premotor neurons to reach spike threshold earlier, predicting a reduction in response latency on the order of 50 ms. Second, if the fast auditory-evoked neural response (<50 ms) to call playback leads to a strong enough depolarization, it can lead to premotor spiking activity even before the arrival of production-related input. Whether this auditory-evoked activity is sufficient to trigger a call response in vivo remains to be investigated.

One prerequisite for the model’s replication of in vivo recorded activity of HVC neurons during calling is an excitatory “vocal-related” input to HVC occurring at the onset of call production–related changes in activity. This raises the question: What is the source of excitation that would drive an increase in interneuron activity and causes premotor neurons to burst?

For calls that are produced in response to the heard calls of conspecifics, afferent auditory-related input onto HVC would be one likely source. It is known that premotor nucleus HVC receives excitatory input from multiple areas: The thalamic nucleus Uvaeformis sends both vocal- and auditory-related information to HVC (35, 62, 63). Sensorimotor nucleus NIf provides the largest source of auditory information onto HVC premotor neurons and interneurons (37, 64, 65) (reviewed in ref. 51), and there is evidence of direct auditory input from other regions of the auditory forebrain as well (66).

Although there is some evidence for direct input from auditory forebrain areas field L and the lateral caudal mesopallium (CM) (66), NIf appears to be a likely candidate area for several reasons. NIf projects directly onto HVC and provides its strongest source of auditory information (51, 53, 67). The time course of activity of the predicted vocal-related input population in relation to the onset of calls (Fig. 1*D*) closely matches that of neurons previously recorded in NIf during call production (52). The timing of call production–related NIf activity relative to call production–related activity in HVC is consistent with monosynaptic inputs. Similar timing of song-related NIf premotor activity has been reported in zebra finches and Bengalese finches (34, 68, 69).

While call production–related increase in interneuron activity necessitates an excitatory drive, premotor bursts could hypothetically be a result of postinhibitory rebound depolarization. However, this phenomenon appears to be absent in most premotor neurons in adult zebra finches (44, 70, 71), reducing the likelihood that premotor bursts were triggered solely by the offset of inhibition, without any excitatory input. Another excitatory neuron type in HVC that projects to “area X” of the basal ganglia does exhibit rebound spiking. These cells sparsely synapse onto premotor neurons (31) and could thereby theoretically induce premotor bursts in a scenario in which external excitation only drives interneurons (70). Interneurons, however, do not return to their baseline firing rate until after call production onset (20.9 ± 19.9 ms), which is after the average burst onset of premotor cells (−14.4 ± 23.8 ms). Thus, the relative timing of premotor and interneuron activity and the sparse connectivity profile between HVC-X neurons and premotor neurons does not support rebound spiking-induced excitation as a mechanism for premotor drive.

It is important to note that we modeled a single hypothetical bursting premotor neuron, which we assume to be representative for the entirety of premotor neurons. The recorded activity among the different premotor and interneurons was qualitatively similar: Sparse bursts and a transient increase in firing rate, respectively (17). Each individual neuron exhibited a relatively stereotyped time course across trials, with respect to call production onset. Across neurons, however, the timing differed for both premotor and interneurons (17) (Figs. 1*D* and 5*B*). Similar variability in the timing of vocal-related input neurons could account for these observations. Subsets of these neurons that ramp up in activity at different time points could thus drive different subsets of HVC premotor and interneurons that become active at different time points relative to call production onset.

In conclusion, the model we propose allowed us to examine social coordination from the perspective of a relatively simple sensorimotor circuit and has highlighted several potentially important mechanisms. Specifically, vocalization-related premotor inhibitory strength can achieve temporal fine tuning of vocal output and auditory-evoked inhibition can transiently suppress premotor drive, thereby reducing simultaneous calling, i.e., jamming. The role of inhibition, in both of these regulatory processes, is more extensive than previously thought and suggests that further investigation of inhibitory cell types and connectivity is required within the songbird vocal–motor pathway and other sensorimotor circuits more broadly. The underlying feedforward wiring scheme of excitatory and inhibitory neurons can be found across brain areas and species. Applying this model to the study of vocal turn taking in other experimentally tractable model systems, including singing mice (72) and marmosets (73–75), would determine whether these mechanisms are general inhibitory principles of interactive vocal control. Our model therefore provides a versatile framework for testing predictions about vocal turn-taking behaviors observed across a variety of time scales and species.

## Materials and Methods

### Animals and Electrophysiological Recordings.

All animal care and experimental procedures were performed with the ethical approval of the Max Planck Institute for Ornithology and the Regierung von Oberbayern (ROB-55.2–2532.Vet_02-18-182). Birds were acquired from the breeding facility at the Max Planck Institute for Ornithology. Birds were maintained in a temperature- and humidity-controlled environment with a 14 h/10 h light/dark schedule and ad libitum food and water. Extracellular HVC recordings were performed with a 16-channel silicon probe (NeuroNexus) in four awake head-fixed adult male zebra finches (>90 d posthatching). Intracellular recordings in NIf were performed with sharp glass pipette electrodes in six awake head-fixed adult male zebra finches. Data for intracellular microdrive recordings in HVC in awake-behaving birds were obtained from recordings reported in ref. 17.

### Data Analysis and Model Neuron Simulations.

We used Plexon Offline Sorter for spike detection and clustering and MATLAB R2020a and Python 3.7 for data analysis. Model simulations were carried out in Python 3.7 using Brian 2 version 2.2.2.1. To simulate the membrane potential dynamics of neurons in the zebra finch song system, we used a leaky integrate-and-fire neuron model with current-based synapses. Model neurons between the different populations are connected randomly in an all-to-all manner, with connection probabilities given in *SI Appendix*, Table S2.

## Supplementary Material

Supplementary File

## Data Availability

Detailed information for the materials and methods used in this study is provided in *SI Appendix*, *Materials and Methods*. Electrophysiological, behavioral, and modeling data, and code data have been deposited in Github (https://github.com/nortonph/call-timing). All other study data are included in the article and/or supporting information.
