# Supplementary material for: A feedforward inhibitory premotor circuit for auditory–vocal interactions in zebra finches
Source: Proc Natl Acad Sci U S A. 2022 Jun 3;119(23):e2118448119. doi: 10.1073/pnas.2118448119 (PMC9191632; doi:10.1073/pnas.2118448119)
Supplement: Supplementary File [file pnas.2118448119.sapp.pdf]

PNAS
www.pnas.org

## Supplementary Information for

*A feed-forward inhibitory premotor circuit for auditory-vocal interactions in zebra finches*

Philipp Norton[1], Jonathan I. Benichov[1], Margarida Pexirra, Susanne Schreiber,
& Daniela Vallentin

[1]These authors contributed equally to this work.

Daniela Vallentin
Email: daniela.vallentin@orn.mpg.de

**This PDF file includes:**

Materials and Methods
Figures S1 to S7
Tables S1 to S2

## MATERIALS AND METHODS

**Animals.** All animal care and experimental procedures were performed with the ethical approval of the Max Planck Institute for Ornithology and the Regierung von Oberbayern (ROB-55.2-2532.Vet_02-18-182). Extracellular recordings were performed in 4 adult male zebra finches (> 90 days post hatching). Intracellular recordings in NIf were performed in 6 adult male zebra finches. Birds were acquired from the breeding facility at the Max Planck Institute for Ornithology. Throughout the experiments, the birds were maintained in a temperature and humidity-controlled environment with a 14/10 hour light/dark schedule and ad-libitum food and water.

**Surgery.** In all cases, zebra finches were anesthetized with isoflurane (1–3% in oxygen). The centers of RA, HVC, and NIf were located based on stereotactic coordinates and small craniotomies and durotomies were performed at the cranial surface targets. A chlorided silver ground wire (0.001", California Fine Wires) was implanted above the cerebellum. For nucleus targeting, a carbon fiber microelectrode (Kation Scientific) was advanced to the coordinates of RA to verify its location via detection of characteristic high frequency tonic multiunit activity. For antidromic identification of HVC-RA projecting premotor neurons, a bipolar stimulating electrode was implanted into RA and antidromic responses were confirmed via the targeting electrode in HVC. For antidromically identifying NIf-HVC projecting neurons, the stimulating electrode was relocated to HVC and correct targeting location in NIf was verified via stimulation in HVC. A custom-made stainless steel head plate was affixed to the skull using dental acrylic (Paladur, Kulzer International). The craniotomies were protected until experiments were conducted using a silicone elastomer (Kwik-Cast; WPI). Animals were returned to their home cage with a companion bird for at least 24 hours post-surgery and were monitored to ensure full recovery before experiments commenced.

**Playbacks.** For measuring neural responses to calls, we presented call playbacks at 65 dB through a speaker placed in front of the head-fixed birds. The presented stimuli were recordings of an average male "stack" call, presented using a custom-made Labview (National Instruments) interface in blocks of 10 at a rate of 1 call per second (1), with 30 seconds of silence between each block. A 10 ms 15 kHz pulse (beyond zebra finch auditory range) was simultaneously played at the onset of each stimulus to ensure subsequent uniform alignment of playbacks with the neural data.

**Electrophysiological recordings.** All recording sites were localized with the following stereotaxic coordinates, with anterior/posterior and medial/lateral positions listed relative to Lambda (the bifurcation of the midsagittal sinus) and dorsal/ventral positions indicating penetration depths, relative to the brain surface. Head angles (HA) as measured from beak tip to ear bar are relative to horizontal and electrode angles (EA) are relative to vertical within a given sagittal plane. HVC: 45° HA, 0° EA, 0.2mm anterior, 2.3mm lateral, 0.15-0.75mm ventral; RA: 45° HA, 35° EA, 1.85mm posterior, 2.25mm lateral, 1.4-2.3mm ventral; NIf (based on (3)): 45° HA, 0° EA, 2.2mm anterior, 1.7mm lateral, 2-2.5mm) ventral.

*Motorized intracellular microdrive recordings in HVC:* Data were obtained from recordings reported in Benichov & Vallentin, 2020 (2). In brief, recordings were performed with sharp glass pipette electrodes (impedance: 70-130 MΩ, backfilled with 3 M potassium acetate) in awake-behaving adult males as they produced calls in response to call playbacks.

*Extracellular recordings in HVC:* during call playback were performed in head-fixed awake birds held in a soft foam restraint. A 16-channel silicon probe (NeuroNexus) was lowered into HVC (between 300-700 µm from the dorsal surface) using a micromanipulator (Sutter Instruments). Neural activity was digitized at a sampling rate of 30 kHz on an Intan RHD2132 headstage and acquired with an RHD Recording Controller (Intan Technologies). A TTL pulse was triggered by the 15 kHz tone at the onset of each playback presentation using an Arduino Uno, and delivered to the RHD Recording Controller for acquisition alongside the neural data.

*Intracellular recordings in NIf:* Awake birds were held in a soft foam restraint and head-fixed. Sharp glass pipette electrodes (impedance: 70-130MΩ) were prepared with a horizontal micropipette puller (P-97; Sutter Instruments) and backfilled with 3 M potassium acetate. NIf was targeted based on stereotaxic coordinates (3) using a micromanipulator (Sutter Instruments). In the vicinity of NIf, the pipette was advanced in ~5µm steps until positioned against a somatic membrane, as indicated by an increase in electrode resistance. A brief (~10 ms) “buzz” pulse was then delivered to penetrate the membrane. Once a stable recording (spike height: > 40 mV, resting membrane potential: < -50 mV, recording duration: > 3 min) was achieved, call playbacks were presented. Membrane potential signals were amplified (Neuro Data IR183A, Cygnus Technology, Inc.), digitized at 40 kHz using a National Instruments acquisition interface, and recorded with custom MATLAB software. HVC-projecting neurons were identified by stimulating ipsilateral HVC with single biphasic current pulses (200 µs per phase, <250 µA) and detecting reliable, stereotypically time-locked antidromic spikes in the NIf cell.

**Data Analysis.** We used Plexon Offline Sorter for spike detection and clustering and MATLAB R2020a and Python 3.7 for data analysis. For the analysis of the extracellular recordings (Figure 3) only neurons were regarded that had a minimum of 20 trials (i.e. playbacks).

Spike rate time series in Figures 1D and 4A & C were calculated with a bin size of 5 ms and smoothed using a Savitzky-Golay filter with window length 9 and polynomial of order 3. Spike rate time series in Figure 3 were calculated with a bin size of 11.1 ms, linearly interpolated to a 1 ms resolution and then smoothed using a Savitzky-Golay filter with window length 99 and polynomial of order 2.

Significant responses of the extracellularly recorded HVC neurons (Figure 3) were determined as follows: Responses were defined as periods in which the average spike rate ± SEM after call playback onset crossed a threshold of two standard deviations above/below baseline firing rate remained above/below this threshold for at least 15 ms. If two positive or negative response onsets followed each other within a time interval of 200 ms, the two responses were merged and counted as one response starting at the onset of the first and ending at the offset of the second response. If the gap between two positive or negative responses was shorter than 120 ms, then this time interval was extended to 350 ms.

One of the eight intracellularly recorded interneurons was omitted from the analysis (Figure 1D and corresponding values), due to the low number of trials (n=3). Its activity peaked at 59.2 Hz, 2.5 ms after call onset and returned to baseline at 71.6 ms.

**Neuron model.** To simulate the membrane potential dynamics of neurons in the zebra finch song system, we used a leaky integrate-and-fire neuron model with current-

based synapses. The voltage dynamics of the membrane are described by the equation

$$\tau_m \frac{dv}{dt} = E_L - v + R_m (I_e + I_s), \quad (1)$$

where $v$ is the membrane potential, $E_L$ the leak potential (or resting potential), $R_m$ the membrane resistance, and $\tau_m$ the membrane time constant. When the membrane potential of a neuron reaches its threshold $v_{thresh}$, it is instantaneously set to its reset potential $v_{reset}$ and a spike is emitted. $I_e$ and $I_s$ are the electrode current and synaptic current, respectively. $I_e$ is used to inject either a time-varying current into the predicted upstream populations (Figure S2) or a small constant current representing unspecific background excitation. Synaptic currents are determined by:

$$\tau_1 \frac{dI_s}{dt} = \frac{\tau_2^{\frac{\tau_1}{\tau_2 - \tau_1}}}{\tau_1} * s - I_s, \quad (2)$$

$$\tau_2 \frac{ds}{dt} = -s, \quad (3)$$

where $\tau_1$ is the decay time constant ($\tau_{decay}$) and $\tau_2$ the rise time constant ($\tau_{rise}$) of the bi-exponential synaptic current (Figure S7). Each time a presynaptic neuron spikes, the corresponding synaptic weight is added to $s$ in the postsynaptic neuron.

Parameters for excitatory and inhibitory model neurons and synapses were fit to data from electrophysiological studies of zebra finch $HVC_{(RA)}$ premotor neurons and HVC interneurons, respectively (4-6), and are given in Table S1. As such studies are sparser for nuclei upstream of HVC, and as we don't know the exact source of the excitatory input we propose, we chose to use the same parameters that were fit to HVC premotor neurons for the predicted upstream populations ("excitatory" in Table S1).

**Input currents.** Neurons in the predicted upstream populations (vocal- and auditory-related input) are driven by a time-varying input current that is aligned to the onset of call production or playback onset, respectively. For the vocal-related input neurons, these currents are characterized by a constant baseline current, followed by a quadratic upstroke from 80 to 10 ms prior to call onset and a linear return to baseline from 10 to 0 ms prior to call onset (Figure S2A). Input to the auditory-related input neurons ramps up between 10 and 35ms after playback onset and returns to baseline between 35 and 60 ms (Figure S2B). For the vocal-related input neurons, baseline current is 170 pA and input peaks at 220 pA (auditory-related: 168 pA and 187 pA, respectively).

To induce variability between neurons in their spiking pattern, at each timestep a time-varying offset is multiplied with the current value at that timestep and added to the input current. This time-varying offset changes every millisecond, where a new pseudorandom value is drawn from a normal distribution with mean 0 pA and variance 200 pA (Figure S2). Additionally, each current segment (baseline, upstroke, downstroke, baseline) for each neuron is offset by a pseudorandom value drawn from a normal distribution with mean 0 pA and variance 10 pA. The remaining neural populations (inhibitory and premotor neurons) receive a constant input current of 30 pA that represents unspecific background excitation.

**Network connectivity.** Model neurons between the different populations are connected randomly in an all-to-all manner, with connection probabilities given in Table S2. There are no recurrent connections between neurons within populations.

**Simulation:** Model simulations were carried out in Python 3.7 using Brian 2 version 2.2.2.1 (7). Equations (1–3) were integrated analytically (using Brian's 'exact' method), with a constant time step of 0.02 ms.

Except for the premotor neuron, all model neurons were initialized with different membrane potentials between $E_L$ and $v_{thresh}$, drawn pseudorandomly from a uniform distribution.

For visualization purposes, artificial spikes were added to the model voltage traces in Figures 1, 2, 4 and 5 as vertical lines above spike threshold at the time points of each spike.

**Data and code availability:** Data and code can be downloaded here: https://github.com/nortonph/call-timing

**References:**


1. J. I. Benichov *et al.*, The Forebrain Song System Mediates Predictive Call Timing in Female and Male Zebra Finches. *Curr Biol* **26**, 309-318 (2016).
2. J. I. Benichov, D. Vallentin, Inhibition within a premotor circuit controls the timing of vocal turn-taking in zebra finches. *Nat Commun* **11**, 221 (2020).
3. E. E. Bauer *et al.*, A synaptic basis for auditory-vocal integration in the songbird. *J Neurosci* **28**, 1509-1522 (2008).
4. R. Mooney, J. F. Prather, The HVC microcircuit: the synaptic basis for interactions between song motor and vocal plasticity pathways. *J Neurosci* **25**, 1952-1964 (2005).
5. G. Kosche, D. Vallentin, M. A. Long, Interplay of inhibition and excitation shapes a premotor neural sequence. *J Neurosci* **35**, 1217-1227 (2015).
6. K. Hamaguchi, M. Tanaka, R. Mooney, A Distributed Recurrent Network Contributes to Temporally Precise Vocalizations. *Neuron* **91**, 680-693 (2016).
7. M. Stimberg, R. Brette, D. F. Goodman, Brian 2, an intuitive and efficient neural simulator. *Elife* **8** (2019).

# FIGURES S1-S7

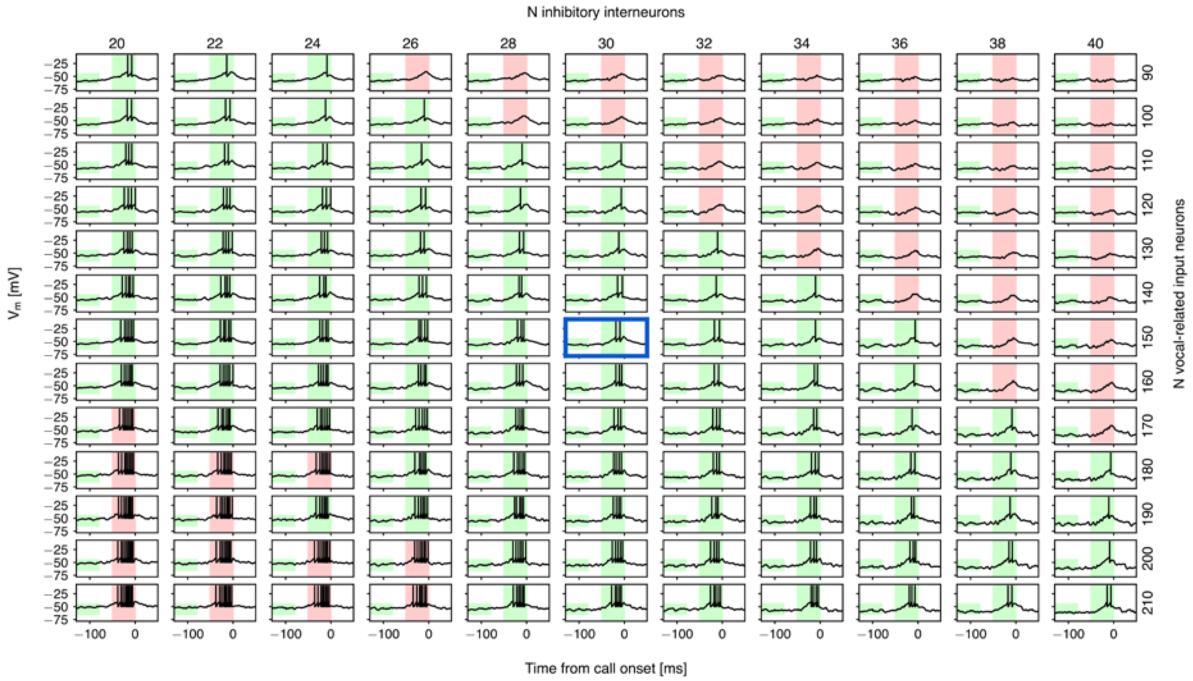


**Figure S1 – Sensitivity analysis for the Feed-Forward Phasic Inhibition model: population sizes.** Membrane potential traces of the model premotor neuron in dependence on the number of inhibitory interneurons (left to right) and vocal-related input neurons (top to bottom). The colored rectangles show whether two criteria are fulfilled (green) or violated (red) in the different simulations: First (left rectangle in each panel), that the baseline membrane potential (average potential between 130 and 80 ms prior to call onset) is in the range of the recorded premotor neurons. This range is between 5 and 25 mV below spike threshold (see e.g. Figure 1E), which corresponds to -65 – -45 mV in the model. Second (right rectangle in each panel), that the neuron produces between one and six spikes during the 50 ms prior to call onset, as was observed in the recorded premotor neurons. The blue frame marks the parameter combination used in the simulations (30 interneurons, 150 vocal-related input neurons).

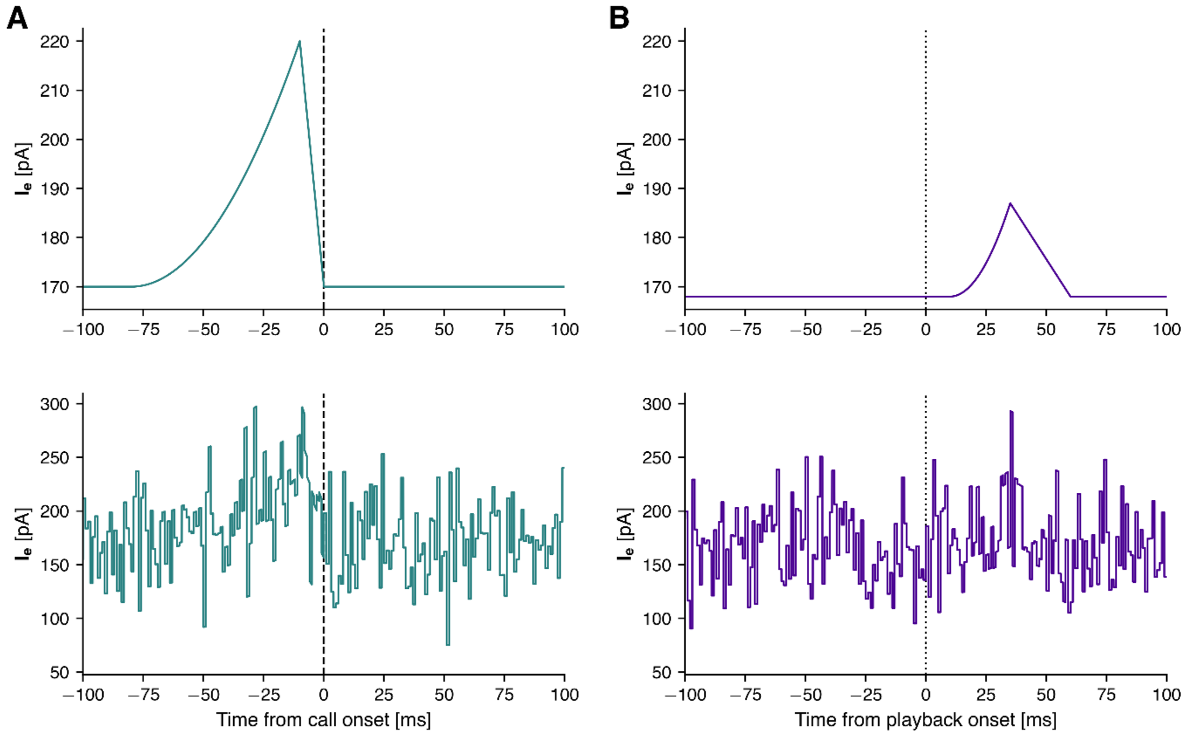


**Figure S2 – Example input currents to the upstream populations.** (**A**) Input current to a neuron in the vocal-related input population, before (top) and after adding a time-varying randomized offset (bottom). (**B**) Same for the auditory-related input population.

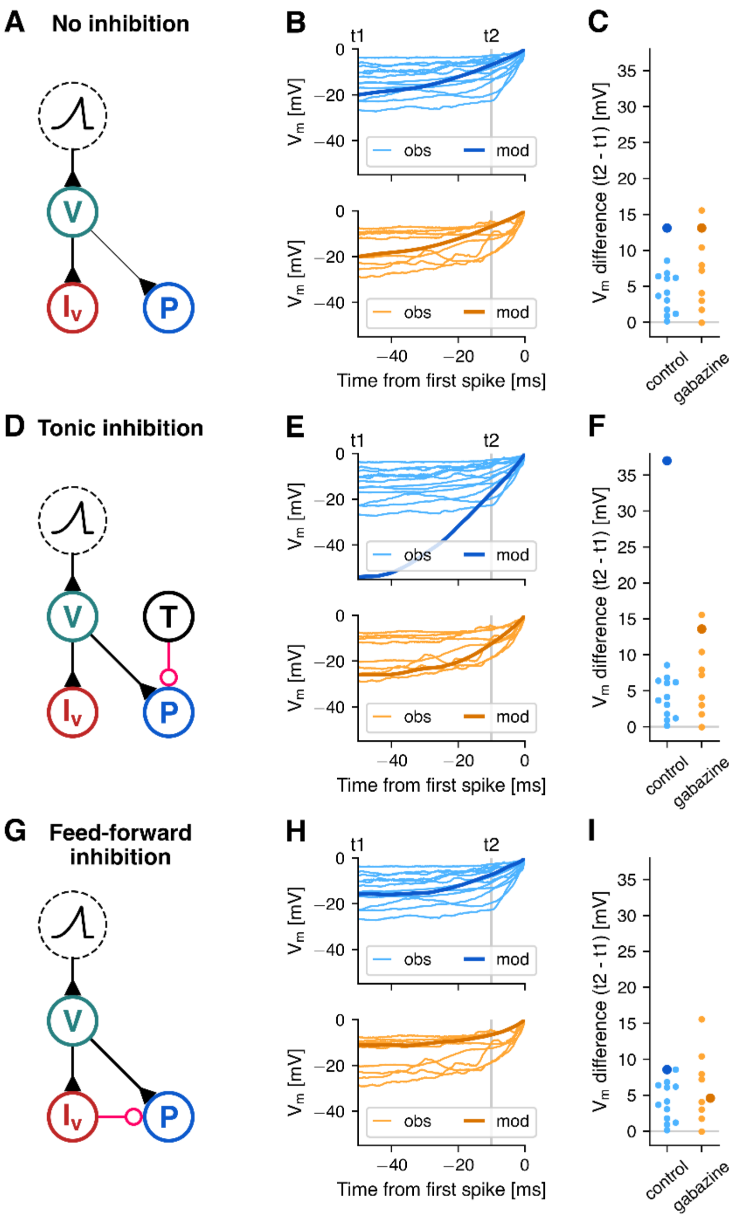


**Figure S3 – Comparison of pre-burst subthreshold potential between different modeled and observed premotor neurons** – (A, D, G) Circuit diagrams of the three model variants introduced in Figure 2. (**B, E, H**) Ramping subthreshold membrane potential of the twelve observed HVC premotor neurons that burst around call onset during the control (light blue, top) and gabazine condition (light orange, bottom), as well as the model premotor neuron at -19pA (dark blue, top) and -7pA inhibitory weights (dark orange, bottom) in the No Inhibition (B), Tonic Inhibition (E) and Feed-Forward Phasic Inhibition (H) models. Traces are aligned to the time point and the membrane potential of their first spike onset (0 ms; 0 mV). Observed traces were averaged across trials and the model traces were averaged across 100 simulations, each with different randomized amplitude offsets in the input current onto the vocal-related input neurons. (**C**, **F**, **I**) Comparison of the differences in membrane potential between 10 ms (t2) and 50 ms before burst onset (t1) for observed (small, light-colored dots) and model premotor neurons (large, dark-colored dots).

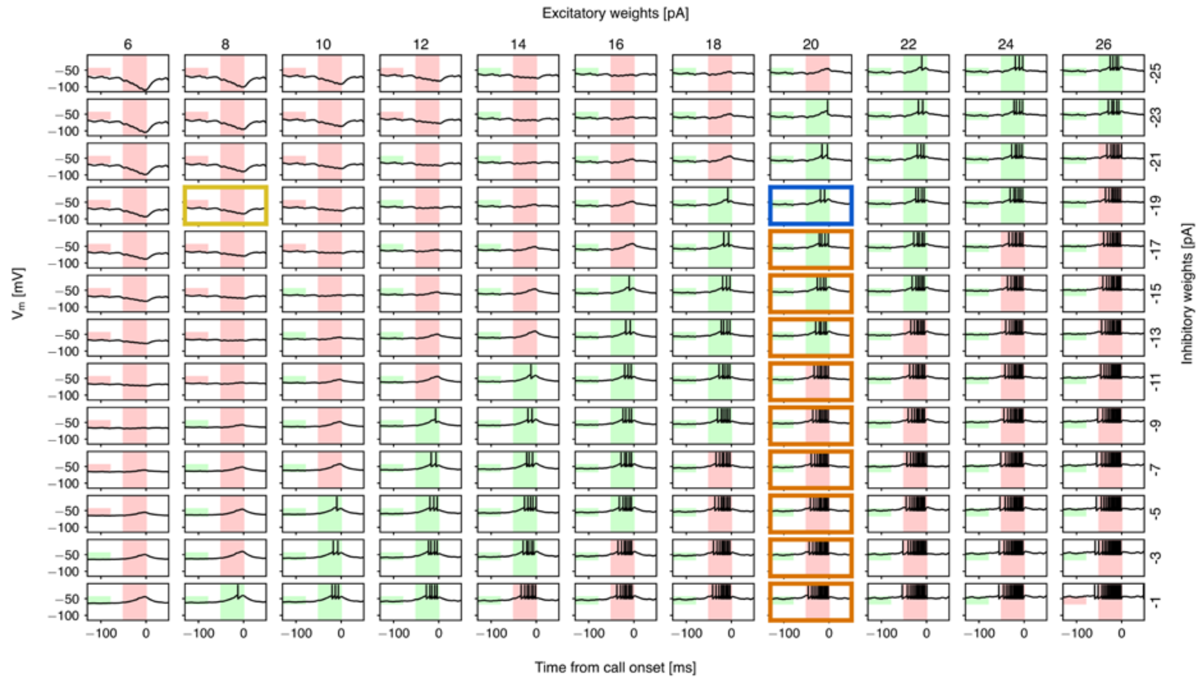


**Figure S4 – Sensitivity analysis for the Feed-Forward Phasic Inhibition model: synaptic weights.** Membrane potential traces of the model premotor neuron in dependence on the weights of its excitatory (left to right) and inhibitory inputs (bottom to top). The colored rectangles show whether two criteria are fulfilled (green) or violated (red) in the different simulations: First (left rectangle in each panel), that the baseline membrane potential (average potential between 130 and 80 ms prior to call onset) is in the range of the recorded premotor neurons. This range is between 5 and 25 mV below spike threshold (see e.g. Figure 1E), which corresponds to -65 – -45 mV in the model. Second (right rectangle in each panel), that the neuron produces between one and six spikes during the 50ms prior to call onset, as was observed in the recorded premotor neurons. Colored frames mark the parameter combinations used in the simulations. Yellow (8 pA, -19 pA): silent premotor neuron; Blue (20 pA, -19 pA): bursting premotor neuron; Orange (20 pA, -17 – -1 pA): reduced inhibitory weights in Figure 2E–F.

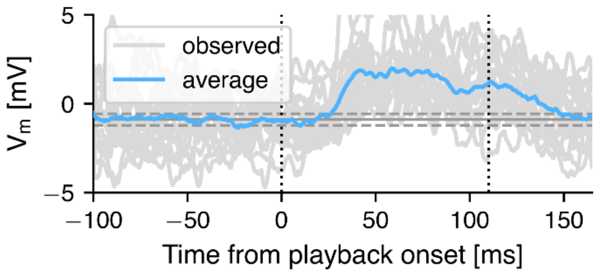


**Figure S5 – Intracellular recordings of an example premotor neuron aligned to playback (dotted lines), which is significantly depolarized following playback onset**. Horizontal lines show mean baseline potential ± 2 standard deviations (baseline: -100–0 ms).

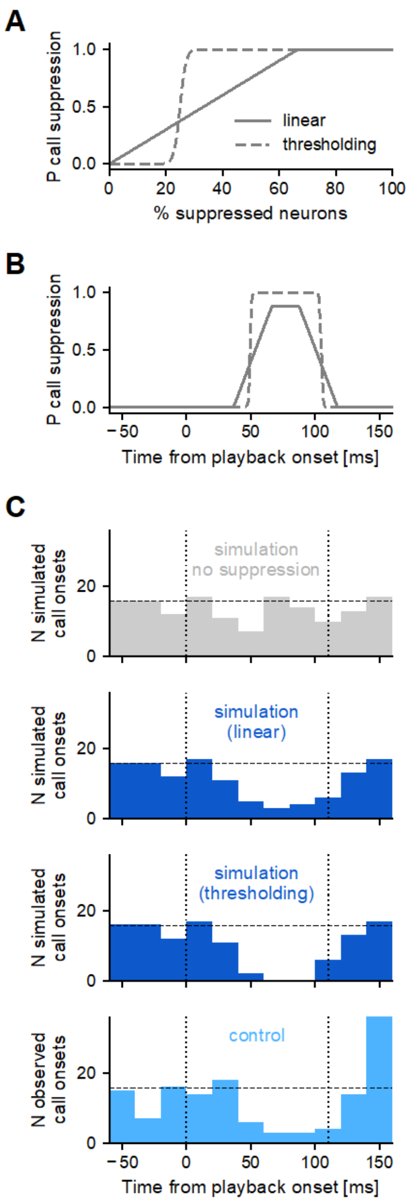


**Figure S6 –Comparison of a linear relationship between number of suppressed premotor neurons and probability of call suppression and a thresholding mechanism.** (**A**) Probability of call suppression (y-axis) in dependence on the percentage of suppressed premotor neurons (x-axis) when assuming a linear relationship (solid line; see Figure 5) or a thresholding mechanism (dashed line) modeled by a logistic function centered on 25% suppressed neurons. (**B**) Probability of call suppression in dependence on time relative to the onset of call playback for both the linear and the thresholding case. This relates the overlap of the two windows (see Figure 5E) with (A). (**C**) Same as Figure 5F but including the simulation results of the thresholding case. In brief: call onsets were removed from a random uniform distribution (top, gray) with the probability given in (B). Experimental data (bottom, light blue; data from Benichov & Vallentin, 2020) shown for comparison.

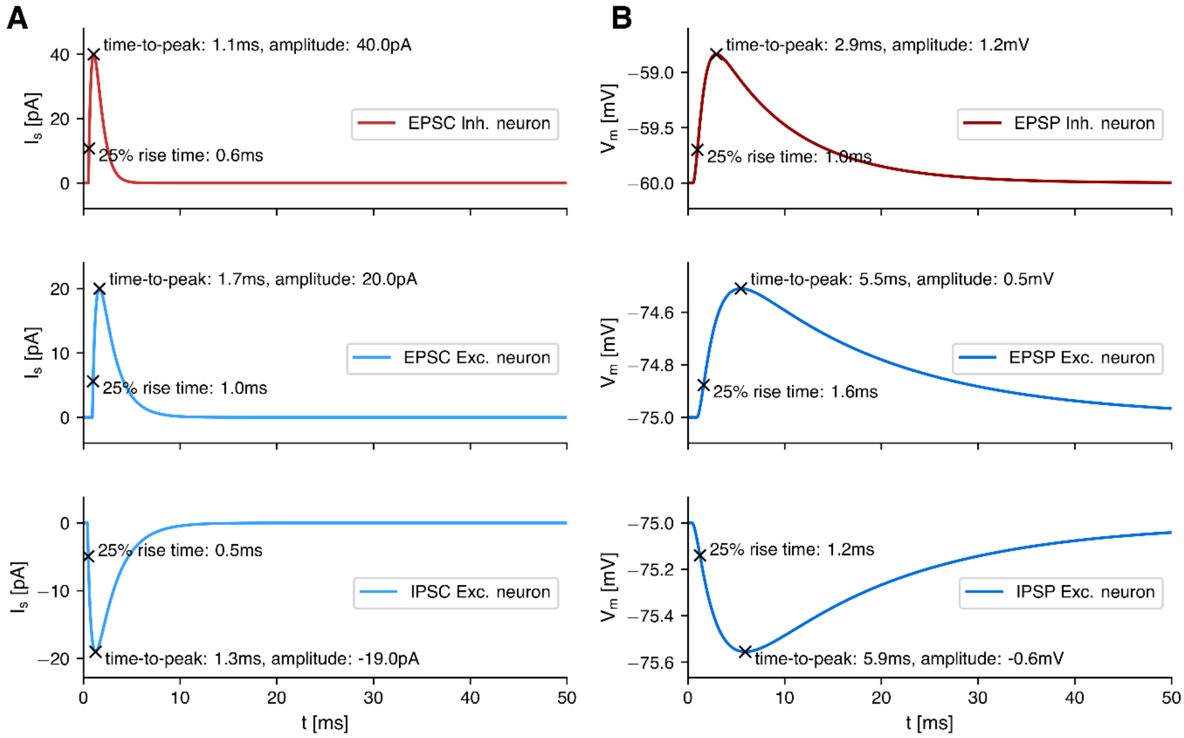


**Figure S7 – Postsynaptic currents and potentials.** (**A**) Excitatory and inhibitory postsynaptic currents (EPSC/IPSC) onto an inhibitory (top) and an excitatory model neuron (middle, bottom) after a single presynaptic spike at time t=0. (**B**) Resulting postsynaptic potentials (EPSP/IPSP) in model neurons at rest.

## TABLES S1 TO S2

**Table S1 - Neuron and synapse model parameters.**

| | $E_L$ [mV] | $v_{reset}$ [mV] | $v_{thresh}$ [mV] | $\tau_m$ [ms] | $R_m$ [MΩ] | $\tau_{decay}e$ [ms] | $\tau_{rise}e$ [ms] | $\tau_{decay}i$ [ms] | $\tau_{rise}i$ [ms] | $t_{ref}$ [ms] |
|---|---|---|---|---|---|---|---|---|---|---|
| **Excitatory** | -75 | -50 | -40 | 16 | 200 | 1.6 | 0.4 | 2.2 | 0.4 | 1 |
| **Inhibitory** | -60 | -70 | -45 | 8 | 200 | 0.6 | 0.5 | 0.6 | 0.5 | 1 |

Parameters used for all excitatory (vocal- and auditory-related input, premotor) and inhibitory neurons (interneurons). $E_L$: leakage or resting membrane potential, $v_{reset}$: reset potential, $v_{thresh}$: spiking threshold potential, $\tau_m$: membrane time constant, $R_m$: membrane resistance, $\tau_{decay}e$/$\tau_{decay}i$: decay time constants for excitatory and inhibitory synaptic currents, respectively, $\tau_{rise}e$/$\tau_{rise}i$: rise time constants for excitatory and inhibitory synaptic currents, respectively, $t_{ref}$: absolute refractory period.

**Table S2 - Network model parameters.**

| Model | No Inhibition (Figure 2A) | Tonic Inhibition (Figure 2C) | FF-Inhibition (Figure 1 & 2E) | Full model (Figure 5A) |
|---|---|---|---|---|
| **Nr. of neurons V** | 150 | 150 | 150 | 150 |
| **Nr. of neurons $I_v$** | 30 | 30 | 30 | 30 |
| **Nr. of neurons P** | 1 | 1 | 1 | 1 |
| **Nr. of neurons A** | — | — | — | 150 |
| **Nr. of neurons $I_a$** | — | — | — | 25 |
| **Nr. of neurons T** | — | 120 | — | — |
| **Conn. prob. V -> $I_v$** | 0.3 | 0.3 | 0.3 | 0.3 |
| **Conn. prob. $I_v$ -> P** | — | — | 1 | 1 |
| **Conn. prob. V -> P** | 0.4 | 1 | 1 | 1 |
| **Conn. prob. T -> P** | — | 1 | — | — |
| **Syn. weights V -> $I_v$** | 40 pA | 40 pA | 40 pA | 40 pA |
| **Syn. weights $I_v$ -> P** | — | — | -19 pA | -21 pA |
| **Syn. weights V -> P** | 20 pA | 20 pA | 20 pA | 20 pA |
| **Syn. weights T -> P** | — | -19 pA | — | — |
| **Syn. weights A -> P** | — | — | — | 10 pA |
| **Syn. delay V -> $I_v$** | 0.5 ms | 0.5 ms | 0.5 ms | 0.5 ms |
| **Syn. delay $I_v$ -> P** | 0.4 ms | 0.4 ms | 0.4 ms | 0.4 ms |
| **Syn. delay V -> P** | 0.9 ms | 0.9 ms | 0.9 ms | 0.9 ms |
| **Syn. delay T -> P** | — | 0.4 ms | — | — |

Parameters used for the different network models presented in Figures 1, 2 and 5. Capital letters denote the different neuron populations. V: Vocal-related input, $I_v$, $I_a$: Interneuron, P: Premotor, A: Auditory-related input, T: Tonically active interneuron. The synaptic weight from the vocal-related input population to the silent premotor neuron (yellow in Figure 1B) was 8 pA instead of 20 pA. Parameters that are identical for all models are shaded in gray. Connection probabilities (Conn. prob.) and synaptic weights and delays between populations A, $I_a$ and P in the full model are the same as between V, $I_v$ and P, except where stated otherwise.
